# Endometrial scratching and intralipid treatment—no general recommendations

**DOI:** 10.3389/frph.2024.1505842

**Published:** 2024-11-27

**Authors:** Paolina Mrosk, Nathallie Sandi-Monroy, Friedrich Gagsteiger, Thomas Wolfram Paul Friedl, Katharina Hancke, Karin Bundschu

**Affiliations:** ^1^ Kinderwunschzentrum Ulm, Ulm, Germany; ^2^ Department of Urology, University Hospital of Ulm, Ulm, Germany; ^3^ Sartorius Stedim Cellca GmbH, Ulm, Germany; ^4^ Department of Gynaecology and Obstetrics, University Hospital of Ulm, Ulm, Germany

**Keywords:** endometrial scratching, endometrial injury, intralipid, uterine natural killer cells, repeated implantation failure

## Abstract

**Objectives:**

Endometrial scratching (ES) and/or intravenous intralipid therapy (in cases of increased uterine natural killer cells, uNKs) are still conducted in several fertility centers as “add-on” treatments in patients undergoing ART, although convincing evidence for beneficial effects is lacking.

**Study design:**

In this retrospective study, associations between ES treatment or additional intralipid therapy and pregnancy and live birth rates of 1,546 patients undergoing 2,821 IVF-/ICSI-treatment cycles with fresh or frozen embryo transfers in a German fertility-center between 1st January 2014 and 31th May 2017 were analyzed.

**Results:**

Overall pregnancy and live birth rates for all 2,821 treatment cycles (468 cycles with ES) were 32.8% and 23.5%. There were no statistically significant differences in pregnancy or live birth rates between first treatment cycles with and without ES (*p* = 0.915 and *p* = 0.577) or between second cycles following an unsuccessful first cycle with and without ES (*p* = 0.752 and *p* = 0.623). These results were confirmed using multivariable generalized estimating equations (GEE) models accounting for non-independency of multiple treatment cycles per patients that included all cycles and showed no significant effect of ES on pregnancy (*p* = 0.449) or live birth rates (*p* = 0.976). Likewise, a GEE model revealed no significant effect of intralipid treatment on pregnancy (*p* = 0.926) and live birth rates (*p* = 0.727).

**Conclusions:**

Our results reveal no evidence that ES increases the pregnancy or live birth rates in women undergoing their first or further IVF cycle with fresh or frozen embryo transfer. Intralipid treatment was also not beneficial. Even if patients explicitly ask for it, these procedures are not recommended outside of clinical studies.

## Introduction

In Germany, between 1997 and 2020 over 364 thousand children were born with assisted reproductive technologies (ART), such as IVF and ICSI, but success rates reached a plateau of approximately 32% per fresh and 30% per frozen embryo transfer (1). Implantation success depends on various factors, like cytokines, interleukins, growth-factors, macrophages and decidualization of stromal cells regulating trophoblast invasion (2, 3). Already in 1907, L. Loeb showed in guinea pigs that endometrial injury - termed endometrial scratching (ES) - increases proliferation of decidual cells (3). ES is a local endometrial trauma caused by a catheter which aspirates mucosal tissue, thereby disrupting the endometrial integrity (4–6). It is assumed that ES leads to an improved synchronization between endometrium and transferred embryo, thereby increasing implantation success (4). However, biological and molecular mechanisms induced by ES are still mostly unknown.

Although Nastri et al. demonstrated an encouraging increase in pregnancy and live birth rates after ES in patients with ART therapy (4), heterogeneous results of other studies challenged the benefit of ES and its applicability in routinely clinical practice (3). Several randomized controlled trials (RCTs) and meta-analyses have now demonstrated the lacking benefit of ES (7–11), but there seems to be a positive effect in certain subgroups (12, 13). ES is still offered and conducted in several fertility centers to patients with recurrent implantation failure (RIF) or miscarriages. Patients often actively ask their physicians for an ES procedure, hoping to increase their pregnancy chances, even if there is no clear evidence for beneficial effects.

Besides ES, other “add-on” treatments such as immune therapies [e.g., intralipids, corticosteroids, Granulocyte-Colony Stimulating Factor (G-CSF) or intravenous immunoglobulin], endometrial receptivity array (ERA), uterine artery vasodilation, and human chorionic gonadotropin instillation) try to improve IVF success rates. However, benefits of these add-ons are often not evidenced yet, and therefore use of these treatments is controversially discussed (14, 15).

For further clarity, we retrospectively evaluated data from a fertility center in Ulm between 1/2014 and 5/2017, when ES treatment was extensively performed due to unclear data at that time. This analysis investigates the impact of ES in patients undergoing ART and compares pregnancy and livebirth rates of patients in the first, second or further fresh or frozen-embryo transfer cycles. Moreover, we analyzed certain subgroups of patients that might benefit from ES. Additionally, we examined potential beneficial effects of intralipid treatment with special attention to patients with enhanced uNK cells detected immunohistochemically in ES samples.

## Materials and methods

Institutional review board (IRB) approval: This study was authorized by the ethics commission of the “Landesärztekammer Baden-Württemberg” (B-F-2017-129).

This retrospective study is based on all ART treatments conducted in “Kinderwunschzentrum Ulm, Einsteinstraße” between 1/2014 and 5/2017. Inclusion criteria were IVF-/, ICSI- fresh or frozen-embryo transfers. Exclusion criteria were multiple pregnancy and missing data of potential previous ES. Stimulation prior fresh embryo transfer was either performed using an antagonist protocol (FSH/LH: Gonal-F®, Puregon®, Bemfola®; Ovaleap®, Pergoveris®, Menogon®; antagonists: Orgalutran®, Cetrotide®) with individually patient adapted FSH-levels or an agonist protocol (FSH/LH as mentioned; down regulation: Synarela® nasal-spray). Frozen embryo transfers were performed in an artificial cycle (oral Estrifam® stepup-protocol until ≥8 mm endometrial size, luteal phase induction with progesterone (Utrogest®, Lutinus®, Progestan®, Famenita®, Prolutex®). Fresh or frozen embryo transfers were performed at day 2/3 in the 4-/8-cell-stage or at day 5 at blastocyst-stage. Following a detailed patient informed consent, ES was performed either in the luteal phase of the pre-cycle of IVF/ICSI or in the mid-luteal phase of the cycle prior frozen-transfer. Endometrial tissue was extracted with a “Pipelle de Cornier” catheter in an outpatient setting in a fertility center in Ulm (city in the South of Germany) and tissue samples were examined histologically in Ulm and Jena (laboratories in two cities in Germany). The immunohistochemical analyzes of uNK cells (CD56-positive) were performed in Jena.

Patients previously identified with increased uNK cells (>300 uNK cells/mm^2^) were offered an off-label intralipid infusion therapy. After written informed consent, patients received intralipid intravenously (50 mL intralipid 20% in 100 mL physiological sodium chloride solution, over 1.5–2 h) once in the previous menstrual cycle, another application at the day of egg retrieval and, if applicable, with a positive pregnancy test.

Descriptive statistics included absolute and relative frequency, mean, standard deviation (SD), median, interquartile range, minimum (min) and maximum (max). Associations between categorical variables and differences between patients with and without ES regarding pregnancy and live birth rates were analyzed by chi-square test. If expected frequencies in cells of 2 × 2 crosstabs were less than five, Fisher's exact test was used instead of chi-square test. Differences between patients with and without ES regarding quantitative data were analyzed with Mann Whitney *U*-test. The effect of ES on pregnancy and live birth rates was analyzed separately for the first treatment cycle of each patient received within the study period, for second treatment cycles following an unsuccessful first treatment cycle, and for third treatment cycles following two unsuccessful treatment cycles. Furthermore, we used Generalized estimating equations (GEE) models (an extension of generalized linear models) to be able to incorporate all treatment cycles into the analyses despite the fact that repeated treatment cycles for the same patient are not independent events. We ran binary logistic models with the dependent variables pregnancy (yes/no) or live birth (yes/no), specifying a binomial probability distribution with a logit link function and incorporating patient ID as subject effect for a repeated measures design. Statistical models for assessment of the effect of ES on pregnancy and live birth rates included ES (yes/no), embryo transfer type (fresh/frozen), patient age (years), patient BMI (kg/m^2^) and partner age (years) as independent predictor variables. Statistical models for assessment of the effect of intralipid therapy on pregnancy and live birth rates (only cases with ES) included only intralipid therapy (yes/no) as independent predictor variable.

A significance level of *α* = 0.05 was used throughout and - given that the analyses presented here are part of a retrospective explorative study - no adjustments for multiple testing were made.

## Results

**Figure 1:**
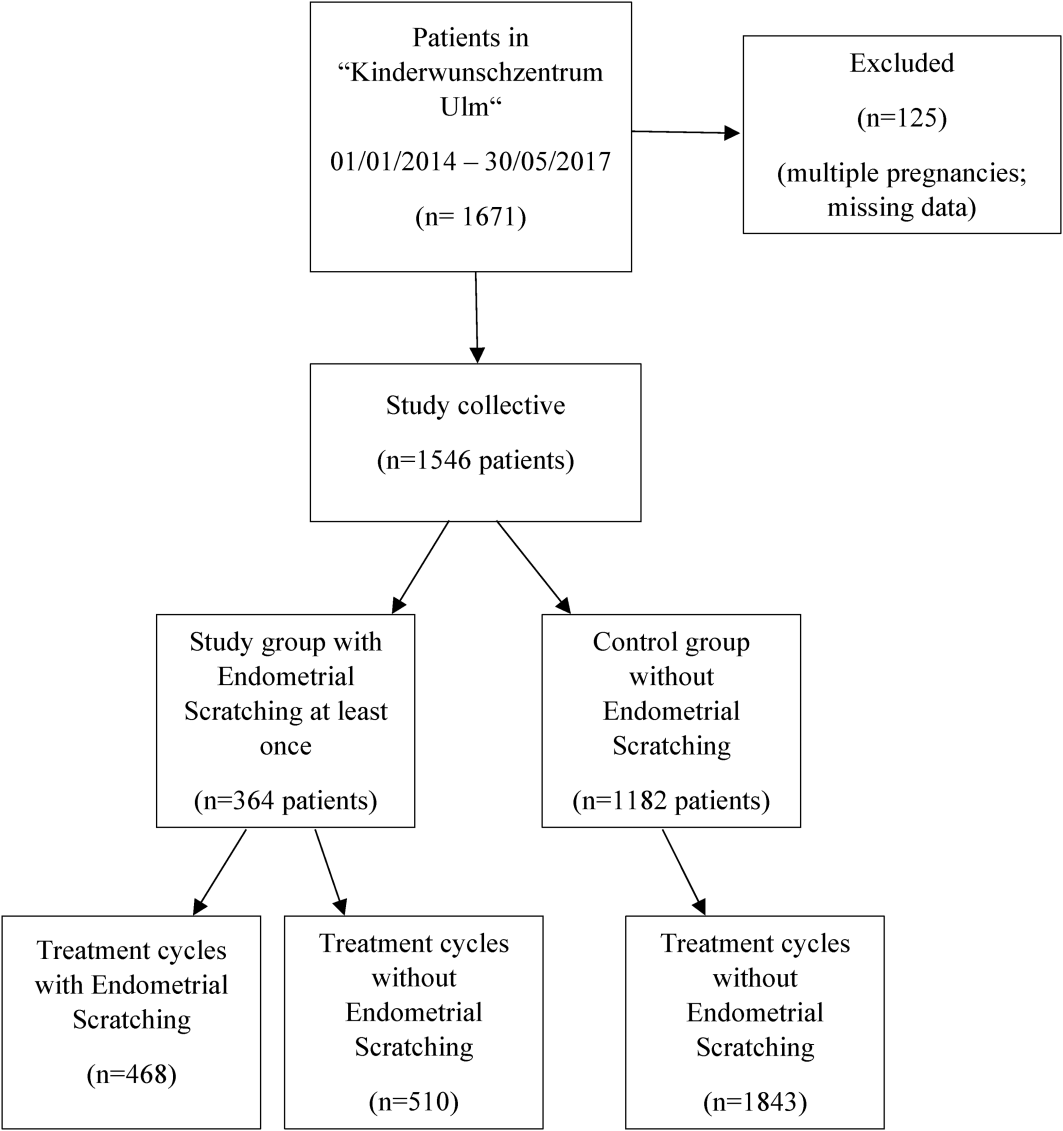
Flow chart of patients included for participation in this study.

Overall, data of 1,671 patients were collected and 1,546 patients were finally included. Of those, more than 50% were older than 35 years, 58% had no previous pregnancy and 70% had not given birth before. 1,546 patients underwent 2,821 treatment cycles (median 1 treatment cycle, range 1–10 treatment cycles) with more than 90% receiving less than four treatment cycles. In total, 364 (23.5%) patients were subject to at least one ES procedure during the study period (range 1–6 treatment cycles with ES), while in 1,182 (76.5%) patients ES was never applied (Figure 1). Table 1 compares the baseline patient characteristics at the first ART treatment. Patients with at least one ES procedure were comparable to patients without ES regarding age, partner age, BMI, gravida, parity and anti-Müllerian hormone (AMH), but had significantly more treatment cycles, with 26.1% vs. 4.0% of patients with more than three treatment cycles overall (Table 1).

**Table 1:** Baseline characteristics for 1,546 patients that received at least one ART treatment during the study period.

Variable	Total *N* = 1,546	No ES *N* = 1,182	ES at least once *N* = 364	*P* ^a^
Maternal age (years)				0.133^b^
Mean ± SD	34.8 ± 4.5	34.7 ± 4.6	35.1 ± 4.2	
Median	35.0	35.0	35.5	
Interquartile range	32–38	31–38	32–38	
Range	22–47	22–47	23–44	
Maternal age (years) - categories				0.062^c^
≤35 years	839 (54.3%)	657 (55.6%)	182 (50.0%)	
>35 years	707 (45.7%)	525 (44.4%)	182 (50.0%)	
Paternal age (years)				0.212^b^
Mean ± SD	38.2 ± 6.1	38.1 ± 6.2	38.5 ± 6.0	
Median	38.0	38.0	38.0	
Interquartile range	34–42	34–42	34–42	
Range	20–89	20–89	26–60	
Maternal BMI (kg/m^2^)				0.962^b^
Mean ± SD	24.5 ± 5.0	24.5 ± 5.0	24.5 ± 5.1	
Median	23.4	23.4	23.4	
Interquartile range	21.0–26.4	21.0–26.3	21.0–26.6	
Range	15.4–63.6	15.4–63.6	16.9–47.0	
Maternal BMI (kg/m^2^) - categories				0.458^c^
Underweight (<18.5 kg/m^2^	43 (2.8%)	29 (2.5%)	14 (3.8%)	
Normal weight (18.5–24.9 kg/m^2^)	926 (59.9%)	715 (60.5%)	211 (58.0%)	
Overweight (25.0–29.9 kg/m^2^)	332 (21.5%)	257 (21.7%)	75 (20.6%)	
Obese (≥30.0 kg/m^2^)	188 (12.2%)	141 (11.9%)	47 (12.9%)	
Unknown	57 (3.7%)	40 (3.4%)	17 (4.7%)	
Gravida				0.967^c^
0	896 (58.0%)	680 (57.5%)	216 (59.3%)	
1	379 (24.5%)	287 (24.3%)	92 (25.3%)	
2 or higher	214 (13.8%)	164 (13.9%)	50 (13.7%)	
Unknown	57 (3.7%)	51 (4.3%)	6 (1.6%)	
Parity				0.062^c^
0	1,086 (70.2%)	808 (68.4%)	278 (76.4%)	
1	345 (22.3%)	275 (23.3%)	70 (19.2%)	
2 or higher	58 (3.8%)	48 (4.1%)	10 (2.7%)	
Unknown	57 (3.7%)	51 (4.3%)	6 (1.6%)	
AMH (ng/mL)				0.577^b^
Mean ± SD	3.11 ± 2.94	3.09 ± 2.91	3.17 ± 3.02	
Median	2.29	2.31	2.25	
Interquartile range	1.05–4.20	1.00–4.20	1.15–4.23	
Range	0.01–15.07	0.01–15.07	0.06–15.00	
Number of treatment cycles received during the study period				<0.001^b^
1	833 (53.9%)	732 (61.9%)	101/27.7%)	
2	401 (25.9%)	308 (26.1%)	93 (25.5%)	
3	170 (11.0%)	95 (8.0%)	75 (20.6%)	
4	77 (5.0%)	33 (2.8%)	44 (12.1%)	
5	40 (2.6%)	8 (0.7%)	32 (8.8%)	
6	13 (0.8%)	4 (0.3%)	9 (2.5%)	
7	8 (0.5%)	2 (0.2%)	6 (1.6%)	
8	3 (0.2%)	0 (0.0%)	3 (0.8%)	
9	0 (0.0%)	0 (0.0%)	0 (0.0%)	
10	1 (0.1%)	0 (0.0%)	1 (0.3%)	

ES, endometrial scratching; AMH, anti-Müllerian hormone.

^a^ Without missings.

^b^ Mann Whitney *U*-test.

^c^ Chi-Square test.

Out of the 2,821 treatment cycles conducted during the study period, 468 (16.6%) included an ES procedure. Table 2 compares the basic features of the single treatment cycles between treatment cycles with and without ES, showing similar parameters regarding the proportion of fresh or frozen embryo transfers, number of fertilized oocytes, frozen fertilized oocytes and embryos transferred per evaluated cycle.

**Table 2:** Basic features for 2,821 ART treatment cycles according to the use of endometrial scratching.

Variable	All treatment cycles (*N* = 2,821)	Treatment cycles without ES (*N* = 2,353)	Treatment cycles with ES (*N* = 468)
Type of embryo transfer			
Fresh	1,798 (63.7%)	1,486 (63.2%)	312 (66.7%)
Frozen	1,023 (36.3%)	867 (36.8%)	156 (33.3%)
Number of fertilized oocytes			
Median	3	3	4
Interquartile range	2–5	1–5	2–4
Range	0–26	0–26	0–16
Number of frozen fertilized oocytes			
Median	0	0	0
Interquartile range	0–0	0–0	0–1
Range	0–19	0–19	0–12
Number of transferred embryos			
Median	2	2	2
Interquartile range	1–2	1–2	1–2
Range	0–3	0–3	1–3

Fresh or frozen embryo transfers were mostly performed at day 2/3 in the 4-/8-cell-stage or as blastocysts at day 5. In fresh embryo transfer cycles, transfer rate at day 5 was 61.5% (192 of 312) with ES and 59.5% (884 of 1,486) without ES. In frozen embryo transfers, day 0 or 5 transfer rate was 80.8% (126 of 156) with ES and 78.4% (680 of 867) without ES.

**Figure 2:**
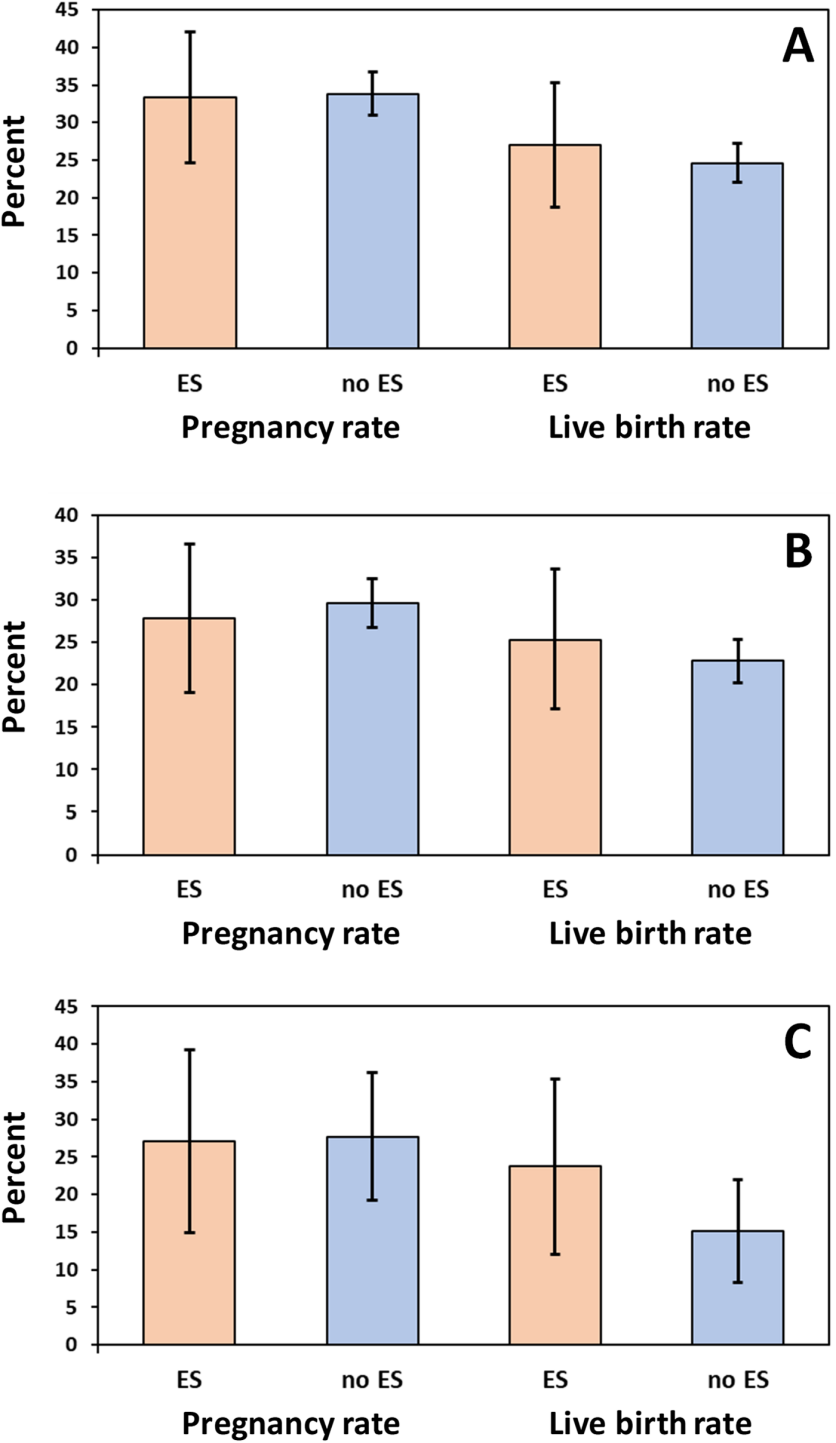
Pregnancy and live birth rates (%) in **(A)** first treatment cycles (*n* = 1,175), **(B)** second treatment cycles following a first unsuccessful treatment cycle (*n* = 457), and **(C)** third treatment cycles following a first and second unsuccessful treatment cycle (*n* = 178). ES, endometrial scratching. Error bars show 95% confidence intervals.

Overall, 1,175 of the 2,821 treatment cycles represent the first ART cycle of a patient, of which 111 (9.4%) and 1,064 (90.6%) were conducted with and without ES, respectively. (Some patients had received their first ART cycle outside the study and the exact number of the treatment cycle was unknown for 351 out of the 2,821 included treatment cycles). Pregnancy rate in the first treatment cycle was 33.3% (95% CI 24.6%–42.1%) with ES and 33.8% (95% CI 31.0%–36.7%) without ES, showing no significant difference (*p* = 0.915; see Figure 2A). Subgroup analysis for fresh or frozen embryo transfers could also not reveal any benefit for patients undergoing ES (Table 3). Likewise, there was no significant difference in the overall live birth rates between first treatment cycles with and without ES (with ES: 27.0%, 95% CI 18.8%–35.3%; without ES: 24.6%, 95% CI 22.0%–27.2%; *p* = 0.577; see Figure 2A); the same result was obtained when live birth rates were compared separately for fresh or frozen embryo transfers (Table 3).

**Table 3:** Pregnancy and live birth rates of women undergoing assisted reproduction in their first treatment cycle.

Variable	First treatment cycle with ES (*N* = 111)	First treatment cycle without ES (*N* = 1,064)	*p*-value
Overall pregnancy rate	37 (33.3%)	360 (33.8%)	0.915^a^
Fresh embryo transfer	31 (32.0%)	281 (33.7%)	0.738^a^
Frozen embryo transfer	6 (42.9%)	79 (34.5%)	0.569^b^
Overall live birth rate	30 (27.0%)	262 (24.6%)	0.577^a^
Fresh embryo transfer	25 (25.8%)	213 (25.5%)	0.955^a^
Frozen embryo transfer	5 (35.7%)	49 (21.4%)	0.203^b^

ES, endometrial scratching.

^a^ Chi square test.

^b^ Fisher's exact test.

Overall, 457 (58.7%) of the 778 patients with one unsuccessful first treatment cycle (no pregnancy) underwent a second cycle during the study course. The pregnancy rate was 27.8% (95% CI 18.0–37.7%) in the 79 patients with ES vs. 29.6% (95% CI 25.0%–34.2%) in the 378 patients without ES (*p* = 0.752). Likewise, live birth rates did not differ significantly between second treatment cycles with or without ES following unsuccessful first treatment cycles (25.3%, 95% CI 15.7%–34.9% vs. 22.8%, 95% CI 18.5%–27.0%; *p* = 0.623; see Figure 2B). As for the first treatment cycle, there were also no significant differences with regard to pregnancy and live birth rates when the comparisons were performed separately for fresh or frozen embryo transfers (Table 4).

**Table 4:** Pregnancy and live birth rates of women undergoing a second assisted reproduction treatment after an unsuccessful first treatment cycle.

Variable	Second treatment cycle with ES (*N* = 79)	Second treatment cycle without ES (*N* = 378)	*p*-value
Overall pregnancy rate	22 (27.8%)	112 (29.6%)	0.752^a^
Fresh embryo transfer	10 (21.3%)	61 (32.1%)	0.141^a^
Frozen embryo transfer	12 (37.5%)	51 (27.1%)	0.230^a^
Overall live birth rate	20 (25.3%)	86 (22.8%)	0.623^a^
Fresh embryo transfer	9 (19.1%)	47 (24.7%)	0.419^a^
Frozen embryo transfer	11 (34.4%)	39 (20.7%)	0.089^a^

ES, endometrial scratching.

^a^ Chi square test.

Overall, 178 (55.1%) of the 323 patients with two unsuccessful treatment cycles (no pregnancy) underwent a third cycle during the study course. The pregnancy rate was 27.1% (95% CI 14.9–39.3%) in the 59 patients with ES vs. 27.7% (95% CI 19.3%–36.2%) in the 119 patients without ES (*p* = 0.931). Likewise, live birth rates did not differ significantly between second treatment cycles with or without ES following two unsuccessful treatment cycles (23.7%, 95% CI 12.0%–35.4% vs. 15.1%, 95% CI 8.3%–22.0%; *p* = 0.159; see Figure 2C). As for the first or second treatment cycle, there were also no significant differences regarding pregnancy and live birth rates when comparisons were performed separately for fresh or frozen embryo transfers (Table 5).

**Table 5:** Pregnancy and live birth rates of women undergoing a third assisted reproduction treatment after two unsuccessful treatment cycles.

Variable	Third treatment cycle with ES (*N* = 59)	Third treatment cycle without ES (*N* = 119)	*p*-value
Overall pregnancy rate	16 (27.1%)	33 (27.7%)	0.931^a^
Fresh embryo transfer	10 (27.0%)	16 (28.6%)	0.871^a^
Frozen embryo transfer	6 (27.3%)	17 (27.0%)	0.979^a^
Overall live birth rate	14 (23.7%)	18 (15.1%)	0.159^a^
Fresh embryo transfer	9 (24.3%)	9 (16.1%)	0.324^a^
Frozen embryo transfer	5 (22.7%)	9 (14.3%)	0.504^b^

^a^ Chi square test.

^b^ Fisher's exact test.

Pregnancy and live birth rates were 34.5% and 25.4% for all 1,486 treatment cycles with fresh embryo transfer but without ES, 29.8% and 22.8% for all 312 treatment cycles with fresh embryo transfer and ES, 31.4% and 21.1% for all 867 treatment cycles with frozen embryo transfer but without ES, and 30.1% and 21.2% for all 156 treatment cycles with frozen embryo transfer and ES.

To account for non-independency of the data (as many patients received more than one treatment cycle), we analyzed the data set including all treatment cycles using an adjusted multivariable GEE model approach (explained in detail in the method section) with the variables ES, type of embryo transfer, patient age, patient BMI and partner age as independent predictor variables for the outcome variables pregnancy rate and live birth rate. There was no significant effect of ES (yes vs. no) on pregnancy rate (adjusted odds ratio 0.92, 95% CI 0.74–1.15, *p* = 0.449) or live birth rates (adjusted odds ratio 1.00, 95% CI 0.78–1.29, *p* = 0.976). Both pregnancy and live birth rate were significantly affected by maternal age at the time of the first treatment cycle received within the study, with decreasing odds for success with increasing age (pregnancy rate: adjusted odds ratio 0.93, 95% CI 0.90–0.95, *p* < 0.001; live birth rate: adjusted odds ratio 0.92, 95% CI 0.90–0.94, *p* < 0.001), indicating that with each year a woman gets older the odds for pregnancy or live birth decrease by 7% to 8%. While there was no significant effect of the type of embryo transfer (frozen vs. fresh) on pregnancy rate (adjusted odds ratio 0.87, 95% CI 0.73–1.03, *p* = 0.101), treatment cycles with frozen embryo transfer were associated with significantly decreased odds for live birth compared to fresh embryo transfers (adjusted odds ratio 0.79, 95% CI 0.65–0.96, *p* = 0.018).

Information on intralipid application was available for 450 of 468 treatment cycles with an ES procedure, and intralipid was applied in 113 (24.1%) of these cases. Pregnancy and live birth rates were 30.1% and 23.9% in the 113 treatment cycles with an ES procedure and intralipid therapy, and 30.6% and 22.3% in the 337 treatment cycles with an ES procedure but without intralipid therapy. A GEE analysis accounting for non-independency of multiple data obtained from the same patient (see methods) showed no significant effect of intralipid (yes vs. no) on pregnancy rates (odds ratio 0.98, 95% CI 0.61–1.57, *p* = 0.926) or live birth rates (odds ratio 1.10, 95% CI 0.65–1.84, *p* = 0.727).

ES tissue samples were examined immunohistochemically for uNK cells in 262 patients and in 79 (30.2%) patients an increase of uNK cells was found, with a mean value of absolute uNK cell number of 413.5 ± 131.8 (median 376 cells, range 304–1,111 cells). Both pregnancy and live birth rates were higher in the 67 patients with increased uNKs cells in ES biopsies and application of intralipid compared to the 12 patients with increased uNKs cells but no subsequent infusion therapy (34.3% vs. 8.3% and 28.4% vs. 0.0%, respectively). However, at least partly due to the small sample size, these differences were not significant (*p* = 0.094 and *p* = 0.060, respectively).

## Discussion

Here, we analyzed retrospectively outcome data of patients treated by ES. Our data revealed no significant differences in pregnancy and live birth rates between patients undergoing ART with ES compared to the control group without ES, even not by considering different subgroups like patient collectives with one or more previous unsuccessful embryo transfers and fresh or frozen embryo transfers. Thus, our large retrospective single-center study adds to the data casting severe doubts on the efficacy of ES, and we are convinced that it is important to make all existing data available to protect patients from potentially unnecessary therapies. Moreover, these therapies can lead to negative side effects, cause extra self-paid costs, are time consuming, promise doubtful false hope while lacking any beneficial effects (11).

In recent years, several RCTs, systematic reviews, and meta-analyses have been published, still reporting controversial results of potentially beneficial effects of ES for improving the reproductive outcomes of infertile women (16, 17). Even if several large RCTs reported no clear evidence for improved live birth rates following ES and the ESHRE add-ons working group reported that ES is currently not recommended for routine clinical use (15), a lot of fertility specialists still offer this procedure to their patients, mostly in cases of recurrent implantation failure (RIF), as demonstrated in a survey study in Australia, New Zealand, and the UK (9, 18).

Our results are in line with the majority of the already published data, which also did not find any significant increase of pregnancy and live birth rates following ES in different contexts (10, 14, 19–24). A recent Cochrane analysis emphasized that even if ES does not affect the chance of miscarriages, it is a slightly painful, unpleasant procedure with small amount of bleeding and in the overall view of available data there is no current evidence to support the routine use of ES for women undergoing IVF (18). Another current Cochrane meta-analysis demonstrated that evidence is insufficient to support ES in women undergoing IUI or attempting to conceive via sexual intercourse (7). Additionally, subgroup analysis of patients with one or two preceding unsuccessful embryo transfers did not show a significant benefit of endometrial scratching in general, which corresponds to data of the current literature (14, 22, 25).

Some previous trials showing a statistically significant benefit of ES were either small or had a higher risk of confounding bias (e.g., missing randomization or overestimation of the treatment effect by early termination of the study) (4, 26, 27). In a meta-analysis, Nahshon and colleges showed improved pregnancy and livebirth rates especially among younger subgroups and in studies that conducted ES twice (28). However, potential confounding factors such as embryo quality were not considered, and they could not prove a significant effect of ES when analyzing studies that solely included patients with two or more previously failed cycles (28), which is in line with our results. Another meta-analysis has shown that ES performed once during the follicular phase of the same cycle of IUI may improve clinical pregnancy rates and further RCTs are suggested on specific populations to ultimately identify the appropriate time of invasion and whether certain subgroups might benefit from ES (12, 29). However, as long as the situation is still unclear, a routinely daily practice of ES should be avoided (15, 22).

Furthermore, in our present study, we could not see any beneficial effects regarding pregnancy or life birth rates of patients treated intravenously with intralipid in cases of increased uNK cells detected in ES biopsies.

Previous studies have shown that abnormal increased expression of NK cells surface markers in peripheral blood, endometrial and uNK cells and higher concentrations of distinct T-lymphocytes are involved in RIF and recurrent miscarriage (RM), assuming that NK cells activity is important for a physiological pregnancy (30–38). In particular, uNK cells seem to play an important role in trophoblast invasion and angiogenesis and represent about 70% of immune cells at the feto-maternal interface (39). However, so far presented data on uNK cells are controversial, not only due to missing standardized diagnostic but also proper reference amounts (40).

Besides ES, immune therapies like intralipid, corticosteroids, Granulocyte-Colony Stimulating Factor (G-CSF) or intravenous immunoglobulin in addition to IVF therapies are widespread and try to improve the success of life birth rates (14). Intralipid is an emulsion of soybean-oil, glycerin egg and phospholipids, usually used as infusion therapy for patients not tolerating oral nutrition. Additionally, intralipid is thought to modulate immune functions and in a mouse model it has been shown to decrease spontaneous abortions (41). In general, intralipid is well tolerated with rare adverse side effects (42), nevertheless complications like allergic reactions, hyperthermia or jaundice have been described (43–45). Depending on the institution, intralipid treatment as “add-on” in ART therapies costs approximately 100–300 US$/€ (14). It was postulated that pre-pregnancy immunomodulatory treatments using intralipid in patients with RM might be helpful for women with abnormal uterine and/or peripheral blood NK cells and establish an immune environment which is supportive for fetal development by recruitment and expression of pro-inflammatory cytokines (34, 36, 46). Singh and colleges have shown in women with previous implantation failure after IVF/ICSI that intralipid increased implantation and live birth rate (47), but the effects are still discussed controversial, and further studies are needed to establish evidence-based guidelines (30, 48). A recently published review and meta-analysis demonstrated that although intralipid is not recommended as a routine treatment for RM or implantation failure, and the presence of abnormal uNK cells as target marker needs further evaluation, a selected patient collective could benefit of intralipid (49). It is assumed, that only patients whose reproductive failure has an identifiable immunologic factor would respond to immunotherapies (50), however the appropriate selection of these subgroups remains challenging.

As valid data are still missing, defining a causal relationship between uNK cells and RIF and RM, as well as a beneficial effect of immunomodulators like intralipid (51–53), immunology testing and treatment outside of well-structured clinical studies is currently not recommended (14). Also the ESHRE add-ons working group remarked that immunomodulating treatments, such as Intralipid, IVIG, rh-LIF, PBMCs, and anti-TNF, are not recommended (15). Although not evidenced, many infertile patients are willing to try anything that might help to increase their chances to get pregnant, despite proven inefficacy, possible side-effects and extra costs for these procedures (54). It also seems to be a psychological problem for infertile couples and for their counselors to discard “potentially useful” naturalized therapies (55).

Of course, we are aware that our retrospective study has its limitations, as it is not a RCT, which is the present gold standard. However, the large size, inclusion of both uNK cell determinations and intralipid therapy, and the incorporation of subgroup analyses with regard to fresh or frozen embryo transfers and treatment cycles following one or two previous unsuccessful ART attempts result in important data that add to the available information necessary to assess the role of ES in ART.

Prospectively, there could be other beneficial effects by further histological, microbiological, or transcriptome analyses of ES material. E.g. detection of an increased amount of uterine plasma cells in chronical endometritis, analyses of a disturbed uterine microbiome, or significant differential gene expression patterns. These might lead to treatments with antibiotics, reconstitution of a physiological endometrial flora, determination of the best time window of endometrial receptivity, or detection of an appropriate secretome profile, respectively (56, 57). Recently, also other approaches were discussed, like intrauterine instillation of growth hormones or autologous platelet rich plasma (PRP) (58). However, all these new methods lack evidence so far.

## Conclusion

Based on our results, there is no clear evidence that ES increases the probability of pregnancy or live birth rates in patients undergoing ART. Even within various subgroup analyses, regarding first or further treatment cycles and fresh or frozen-embryo transfer, we could not detect any clear statistically significant beneficial effects of ES. Furthermore, no positive effect was detectable following an intravenous intralipid therapy. Considering our results together with the majority of the current available literature, we cannot recommend ES and intralipid therapies as valid “add-on” treatment in daily clinical reproductive medicine practice outside of well-designed clinical trials, even if patients might ask their attending physicians for it.

## Data Availability

The original contributions presented in the study are included in the article/Supplementary Material, further inquiries can be directed to the corresponding author.
